# Utilizing Odor-Adsorbed Filter Papers for Detection Canine Training and Off-Site Fire Ant Indications

**DOI:** 10.3390/ani11082204

**Published:** 2021-07-26

**Authors:** Wei-Lien Chi, Ching-Hui Chen, Hui-Min Lin, Chung-Chi Lin, Wang-Ting Chen, Yi-Chen Chen, Yi-Yang Lien, Yi-Lun Tsai

**Affiliations:** 1 Department of Veterinary Medicine, College of Veterinary Medicine, National Pingtung University of Science and Technology, Pingtung 91201, Taiwan; chiita@asia.edu.tw (W.-L.C.); pf.piano@tajen.edu.tw (C.-H.C.); 2 Department of Post-Baccalaureate Veterinary Medicine, College of Medical and Health Science, Asia University, Taichung 41354, Taiwan; 3 Bachelor Degree Program in Pet Care and Grooming, College of Pharmacy and Health Care, Tajen University, Pingtung 90741, Taiwan; 4 Monsters’ Agrotech, Taipei 10088, Taiwan; roar@msagt.com; 5 Department of Biology, College of Science, National Changhua University of Education, Changhua 50007, Taiwan; cclin@cc.ncue.edu.tw (C.-C.L.); a.little@gmail.com (W.-T.C.); 6 Institute of Physics, Academia Sinica, Taipei 11592, Taiwan; stes5813@gmail.com; 7 Research Center of Animal Biologics, National Pingtung University of Science and Technology, Pingtung 91201, Taiwan

**Keywords:** red imported fire ant, *Solenopsis invicta*, odor-adsorbed filter paper, detection dog, aggressive pest

## Abstract

**Simple Summary:**

The red imported fire ant (RIFA, *Solenopsis invicta*) is an exotic pest that can harm humans and animals, cause economic loss to agriculture, and damage ecosystems. In the present study, we devised a practical method to train detection dogs without introducing live RIFAs and an alternative way to correctly identify RIFA-invaded zones. Both live RIFA-experienced and inexperienced detection dogs successfully indicated RIFA-scented filter papers and live RIFAs with a high positive indication rate (>93%) and low false response rate (2%), and also performed successfully in field studies. In addition, the RIFA-scented filter papers can be stored at −20 °C and 4 °C at least 13 weeks for dog identification. Using filter paper as a RIFA odor bearer for detection dog training and RIFA identification is an effective and economical method in order to decrease the risk of RIFA introduction into uninvaded or eradicated areas.

**Abstract:**

The red imported fire ant (RIFA, *Solenopsis invicta*) is an exotic aggressive pest that is notorious for its ability to seriously harm humans and animals, cause economic loss to agriculture, and damage ecosystems. This is the first study to validate the capability of filter paper adsorption as a feasible odor bearer of RIFAs and evaluate its use in detection dog training. Two live RIFA-experienced detection dogs achieved a mean 92% positive indication rate (PIR) on RIFA-scented papers with a relatively low false response rate (0.8%). The similar accuracies in recognizing live RIFAs (96%) and scented papers (92%) suggest that a filter paper is an effective odor reservoir. After training with live RIFA and scented filter papers, both RIFA-experienced and inexperienced detection dogs successfully indicated filter papers that were scented with at least 10 RIFAs for 4 h with a high PIR (>93%) and low false response rate (2%). Detection dogs correctly recognized the filter papers scented by 10 RIFAs for 24 h with a 97.6% PIR. Even for scented samples stored at −20 °C and 4 °C for 13 weeks, the positive indication rates (PIRs) were as high as 90%. These results suggest that filter paper is an effective RIFA odor bearer, and the scent can be maintained at least 13 weeks for dog identification. After RIFA-scented paper training, detection dogs showed high (>95%) PIRs for both RIFA-scented paper and live RIFAs and also successfully performed field studies. Using filter paper as a RIFA odor bearer is an effective and economical method for detection dog training and RIFA identification.

## 1. Introduction

The red imported fire ant (RIFA) *Solenopsis invicta* Buren is an exotic aggressive pest that is notorious for its ability to seriously harm humans and animals, cause economic loss to agriculture, and damage ecosystems [1]. Originally from South America, *S. invicta* invaded the United States in the 1930s and then spread through the southern United States and Caribbean islands [2,3]. In the early 2000s, RIFA territory expanded across the West Indies to the Pacific region; they were first discovered in New Zealand and Australia and later in Taiwan, Hong Kong, Macao, and China [4,5,6,7,8,9].

The ecological and economic impact of RIFA invasion and infestation is enormous [1]. Without strong competitors, fire ants promptly become the dominant ant species in infested regions. The populations of native ants and arthropods are reduced, and native vertebrates such as mammals, birds, and herpetofauna also experienced adverse effects [10]. Immediate burning and itching at sting sites are the characteristic experiences, and hypersensitivity reactions and possibly secondary infections can be very harmful to humans [11,12].

Fire ants, *Solenopsis xyloni*, were first noted to damage electrical equipment in 1940. Telephone failures in Texas were attribute to the ants’ removal of wire insulation and their tendency to nest inside equipment, resulting in excessive internal current flow and short circuits [13]. Over time, problems in highway department signal cabinets and failures in air-conditioning units, telephone services, and traffic equipment were reported with increasing frequency [14,15,16]. RIFAs were later demonstrated to be attracted to electric fields [17].

Besides visual inspection of RIFA mounds, bait traps are the primary method to detect fire ants. Oil baits were tested as attractive chemicals in the 1970s [18,19]. Ali and Reagan (1986) demonstrated that molasses and peanut oil attract RIFAs over short and long exposure periods, respectively [20]. In a monitoring study of RIFAs on a tropical fish farm, cotton cosmetic pads moistened served as a sucrose-based ant attractant [21], while toothpicks dipped in peanut butter were utilized as an oil-based ant attractant [22]. Food lures including heated meat products and corn/potato chips were also used to attract RIFAs and estimate their habitat range and abundance [23,24,25]. While previous access can confirm RIFA appearance, it is less efficient in locating ant nests, especially emerging and small mounds. Moreover, ant detection efficacy using the bait trap method decreases to 30% when RIFA net density is ≤15 nests per ha [22].

Scent-detection dogs are used to detect non-biological scents (e.g., explosives and land mines) and biological scents (e.g., human, animal and plant scents) [26]. They have also been trained to detect destructive insects including the gypsy moth (*Porthetria dispar* L.), red palm weevil (*Rhynchophorus ferrugineus*), screwworm (*Cochliomyia hominivorax* Coquerel), bed bug (*Cimex lectularius* L.), sylvatic triatoma infestans (Hemiptera: Reduviidae), and multiple termite species [27,28,29,30,31,32].

Trained dogs can identify the odor of RIFAs and freely search for nests in the field [33,34]. Our group demonstrated that trained detection dogs still achieved a 93% correct RIFA indication rate even when other ant species were introduced [35]. Dogs show great competence in detecting emerging and smaller RIFA nests in low-density locations [35]. Because of the invasive risks and because live RIFAs may not be available for training, we devised a practical method to train detection dogs without introducing live RIFAs and an alternative way to correctly identify RIFA-invaded zones.

We describe a method using filter paper to adsorb RIFA odors for detection dog training. Filter paper was first tested for its feasibility as an odor bearer of RIFA. The odor concentration and time required for scent adsorption of filter paper were tested, as well as the appropriate storage temperature and time. Dogs trained with odor-adsorbed filter papers were then tested for live RIFA detection and in a field trial.

## 2. Materials and Methods

### 2.1. Canines

Five beagle dogs (one beagle mix) aged 3–7 years old, three females (two spayed) and two neutered males, were tested in this study. They are referred to as Dog A, B, C, D, and E (Table 1). These dogs were family companions and screened for certain characteristics such as self-confidence, physical soundness, food drive, sociability, intelligence, and ability to be trained. After passing the screen for detection dogs, they were trained for odor recognition ability. Dog handlers walked the dogs on leashes and indicated the objects with hiking sticks. The dogs were taught to respond to the RIFA scent by sitting in front of the detection target (passive response). Food and verbal praise were given as positive reinforcements to correct responses to encourage performance. Dogs A and B previously received RIFA training and had acquired the capability to correctly differentiate RIFA, *Solenopsis invicta*, from the other local species of ants, *Crematogaster rogenhoferi*, *Paratrechina longicornis,* and *Pheidole megacephala* [35]. Dogs A and B had served as live RIFA detection dogs for more than 3 years.

### 2.2. Live RIFAs

**Figure 1:**
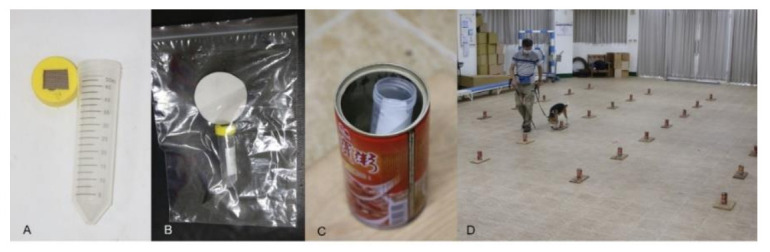
(**A**) A 50 mL centrifuge tube with a 1.7 × 1.7 cm^2^ square copper sieved cap. (**B**) One filter paper and the centrifuge tube with live RIFA inside were put together into a plastic zip bag (28 × 42 cm^2^) and sealed. (**C**) The caps of the tubes with treated or untreated papers were opened one at a time and randomly placed into metal cans for dog detection. (**D**) The scent lineup method was applied in a work room.

RIFAs were collected from mounds in Taiwan as previously described and then cultivated in plastic buckets containing soil [34]. Double-distilled water absorbed in cotton balls was provided, and egg puddings (ingredients mainly eggs, sugar, and milk) and an occasional fried chicken thigh with skin (~13% fat) were supplied as food. Each 50 mL centrifuge tube capped with a 1.7 × 1.7 cm^2^ square copper sieved opening was prepared in a separate sealed bag to avoid non-target odor contamination (Figure 1A). In subsequent experiments, pieces of tissue paper were placed in the buckets to attract ants and then put into reclosable plastic bags that were filled with CO_2_ for anesthesia. The paralyzed ants were then removed into tubes for further testing.

### 2.3. Odor-Adsorbed Filter Papers

Filter paper (No. 2, 90 mm, ADVANTEC Toyo, Ltd., Tokyo, Japan) was used as a scent reservoir. Live RIFAs were placed in a 50 mL centrifuge tube with a 1.7 × 1.7 cm^2^ square copper sieved opening. Then one filter paper and the centrifuge tube with live RIFAs inside were put together into a plastic zip bag (28 × 42 cm^2^) and sealed (Figure 1B). These procedures avoided live RIFAs escaping from the centrifuge tube, but allowed the filter paper to be exposed to the ant scent. The number of ants and adsorption time are described in each section of the different trials. After adsorption, the paper was moved into a new, fully capped and sealed centrifuge tube with forceps and used as a target sample. Unscented filter paper placed in a fully capped and sealed centrifuge tube was used as a non-target sample. All target and non-target samples were placed in a 25 °C incubator for 1 h before testing. Right before the test, the caps of the tubes with treated or untreated papers were opened one at a time and randomly placed into metal cans for dog detection (Figure 1C).

### 2.4. Scent Lineup Method

The scent lineup method was applied in a work room (Figure 1D). Seven to 10 metal cans were lined up at 90 cm intervals, with 210 cm between lines to avoid interference between samples and to supply sufficient space for the handlers and dogs. In a line, only 0–2 metal cans were randomly and discontinuously set as target samples, and the other samples were non-target samples. A blank run (centrifuge tubes with unscented filter paper placed in all metal cans) was performed before each trial start. A double-blind procedure was used; neither the dogs nor their handlers were aware of the positions of the target samples. The handlers were informed if the dog responded correctly by another person who was holding a picture card with Yes/No symbols at the corner of the work room. The dogs were reward by the handler immediately when they responded correctly to the target samples.

### 2.5. Statistical Analyses

The data were analyzed by utilizing SAS Enterprise Guide 6.1 software (SAS Institute, Cary, NC, USA). *P* values less than 0.05 were considered significantly different.

### 2.6. Trial 1: Feasibility of Using Filter Paper to Carry and Represent RIFA Odor

Centrifuge tubes containing 10 live RIFAs inside and tubes with a filter paper scented by 100 live RIFAs for 4 h were prepared as targets; a tube containing untreated filter paper inside was used as a non-target. For each test, we prepared 60 samples including five with 10 live RIFAs, five with 100 live RIFA-scented paper, and 50 samples with untreated paper. Five replicates of the tests were performed by Dogs A and B, which were live RIFA-experienced detection dogs that had not previously been exposed to scented filter paper. Positive indication was defined as a correct response to tubes with live RIFAs/treated filter papers, and a false response was when the dogs responded to untreated filter papers. The average positive indication rate (PIR) was then calculated to determine if filter paper can be used as an odor reservoir and recognized by detection dogs. The PIRs for live RIFAs and scented filter paper were compared with Mann–Whitney U tests.

### 2.7. Trial 2: Odor-Adsorbed Filter Paper—Odor Intensity and Adsorption Time

Two live RIFA-experienced detection dogs (A and B) and three inexperienced detection dogs (C, D, and E) were enrolled in this trial. Filter papers scented by 100 live RIFAs for 4 h were used to train these newly recruited dogs for RIFA odor recognition. The training followed the procedures in our previous publication, “Indoor Training for Red Imported Fire Ant Odor Recognition and Identification” [35]. After 20 consecutive correct responses, dogs were considered to have passed the precursor exercise and were qualified to participate in the following tests.

#### 2.7.1. Dog Detection and Odor Intensity

The target samples were tubes each containing a filter paper scented with 10, 50, or 100 RIFAs for 4 h. A tube with untreated filter paper inside was used as a non-target. For each test, a total of 50 samples were prepared, including 5 samples with scented papers and 45 samples with untreated papers. Five replicates of the tests were performed by each detection dog. The average PIRs in discerning filter paper scented by 10, 50, or 100 RIFAs for 4 h were calculated and compared with Kruskal–Wallis tests.

#### 2.7.2. Dog Detection and Odor Adsorption Time

Target samples were tubes containing filter papers scented by 10 RIFAs for 24 h, 6 h, 1.5 h, 22.5 min, 5.6 min, and 1.4 min (decreased by 4 times). A tube with untreated filter paper inside was used as a non-target. A total of 30 samples were prepared for each test: 6 samples with scented papers and 24 with untreated papers. Five replicates of the tests were performed by each dog. The mean PIRs in discerning filter paper scented by 10 RIFAs for 24 h, 6 h, 1.5 h, 22.5 min, 5.6 min, and 1.4 min were calculated. The association between the odor adsorption time of RIFA-scented filter papers and positive indication rates (PIRs) were tested by calculating Spearman rank correlation coefficients.

### 2.8. Trial 3: Odor-Adsorbed Filter Paper—Storage Time and Temperature

Dogs A, B, and C participated in this trial. The target samples were filter papers scented by 10 RIFAs for 24 h. These samples were prepared and stored at three different temperatures (−20 °C, 4 °C, and 25 °C) for eight storage durations (0 [samples tested on the collection day], 1, 3, 5, 7, 9, 11, and 13 weeks). In total, 24 different conditions were tested. A tube containing untreated filter paper was used as a non-target. For each test, a total of 30 samples were prepared (5 target and 25 non-target samples). Before each test, each sample was placed in an incubator at 25 °C for 1 h. The PIR of each dog and the mean PIR and standard deviation in each condition were calculated. The PIRs were compared using the nonparametric Kruskal–Wallis test. Spearman correlation coefficients were calculated to test the association between storage time and dog PIR, and the data for the three different storage temperatures were analyzed separately.

### 2.9. Trial 4: Odor-Adsorbed Filter Paper Trained Dogs Detecting Live RIFAs

Dogs C, D, and E were inexperienced in live RIFA detection and trained using odor-adsorbed filter paper. After achieving 90% PIR in training, they were qualified to be tested on live RIFA detection. A total of 30 samples were prepared for each test, including one sample of paper scented with 100 live RIFAs, one scented with 10 live RIFAs, one scented with 50 live RIFAs, one scented with 100 live RIFAs, and 26 samples with untreated paper. Each dog completed five rounds with five tests each, and PIRs were calculated. Kruskal–Wallis tests were performed to compare the PIRs of newly trained dogs for the different samples.

### 2.10. Trial 5: Field Trial—RIFA Detection in Culverts

**Figure 2:**
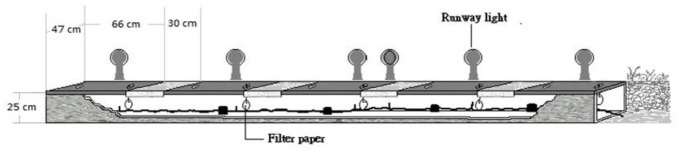
At Taoyuan International Airport, each row of the underground wire culverts below the navigation lights is covered with five steel plates.

The navigation lights at Taoyuan International Airport, Taiwan are above underground wire culverts covered with five steel plates (Figure 2). Nine rows of culverts were selected, and the methods of corn/potato chips attraction and visual mound inspection were used to identify RIFA activity. In each Culvert, 3 uncapped tubes containing potato chips were placed for 2 h. In one row, approximately 200 live RIFAs were identified inside this culvert, but the other eight rows were clear. Then, filter papers were hung by using cotton thread from hand holes under each steel plate to adsorb odor in the nine culvert rows. There were 5 filter papers placed for RIFA scent adsorption in each culvert. After 24 h, the odor-adsorbed filter papers were collected, sealed in separate centrifuge tubes, and sent to the lab at 4 °C in less than 24 h. After incubation at 25 °C for 1 h, the samples were tested by four trained dogs (A, B, C, and E). This trial was practiced twice, and the PIR and false response rates were evaluated.

## 3. Results

### 3.1. Trial 1: Feasibility of Using Filter Paper to Carry RIFA Odor

**Figure 3:**
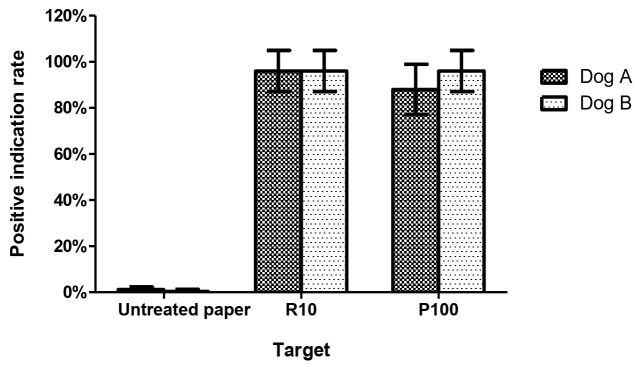
The positive indication rates of two live RIFA-experienced detection dogs (A and B) for detecting 10 live RIFAs in tubes (R10), containing a filter paper scented with 100 live RIFAs (P100) for 4 h, and tubes with untreated paper. The positive indication rate is the mean percentage of positive indications over total indications by dogs. Columns represent the mean and standard deviation of 5 replicates of the tests performed by each detection dog. There were no significant differences between the dogs’ capabilities in distinguishing live RIFAs and RIFA-scented paper (*p* = 0.37, Mann–Whitney U test).

The mean PIRs of two live RIFA-experienced detection dogs (A and B) for detecting 10 live RIFAs in tubes were both 96.0%. After detecting a tube with a filter paper scented by 100 live RIFAs for 4 h, Dogs A and B had PIRs of 88% and 96%, respectively. Their false response rates were 1.2% and 0.4% (Figure 3). The discrimination results were similar for samples of live RIFAs and RIFA-scented papers (*p* = 0.37). The results support the hypothesis that filter paper can be used as an odor reservoir that is recognized by detection dogs.

### 3.2. Trial 2: Odor-Adsorbed Filter Paper—Odor Intensity and Adsorption Time

#### 3.2.1. Dog Detection and Odor Intensity

The five dogs successfully detected filter papers scented by 10, 50, and 100 RIFAs for 4 h. The mean PIRs were 95.2%, 94.4%, and 93.6%, respectively, and the false response rate was 2% (Table 2). There was no significant difference among PIRs for papers scented by 10, 50, and 100 RIFAs (*p* = 0.84).

#### 3.2.2. Dog Detection and Adsorption Time

**Figure 4:**
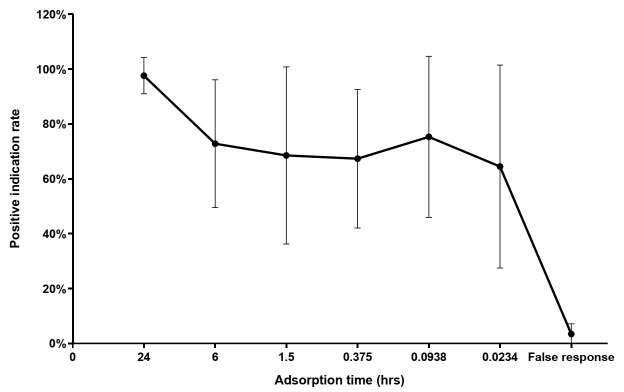
The positive indication rates of five dogs detecting tubes containing filter paper scented by 10 RIFAs for 24 h, 6 h, 1.5 h, 22.5 min, 5.6 min, or 1.4 min. Error bars represent the standard deviation of 5 replicates of the tests performed by 5 dogs (25 pieces of data for each time point). The result showed a weak positive linear relationship between the positive indication rate and filter paper scented time (*r* = 0.29, n = 30, *p* = 0.12; Spearman rank correlation coefficient).

The mean PIR for all five dogs detecting tubes with a filter paper scented by 10 RIFAs for 24 h was 97.6% and tended to decrease with shorter scented time. The PIRs for detecting tubes with a filter paper scented by 10 RIFAs for 6 h, 1.5 h, 22.5 min, 5.6 min, and 1.4 min were between 64.5% and 75.3%, and the false response rate was 3.4% (Figure 4). The non-significant Spearman correlation coefficient value of 0.29 suggests a weak positive linear relationship between the PIR and filter paper scented time (*r* = 0.29, n = 30, *p* = 0.12).

### 3.3. Trial 3: Odor-Adsorbed Filter Paper—Storage Time and Temperature

**Figure 5:**
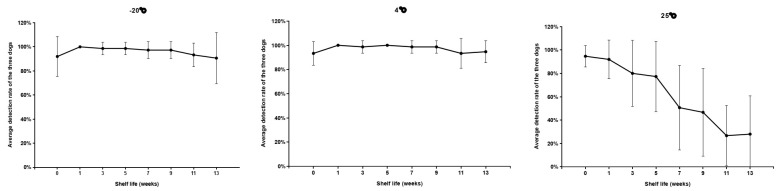
The positive detection rates on the scented filter papers stored in different temperature conditions. Error bars represent the standard deviation of 5 replicates of the tests performed by 3 dogs (15 pieces of data for each temperature condition). The detection rate of samples stored at 25 °C significantly decreased with increasing storage time (*p* < 0.0001, Kruskal–Wallis test) while no differences were found for those stored at −20 °C (*p* = 0.21, Kruskal–Wallis test) and 4 °C (*p* = 0.40, Kruskal–Wallis test).

Three dogs sniffed scented filter papers stored at 24 different temperatures and durations. The samples stored at −20 °C and 4 °C were detectable throughout the 13-week storage period with a >90% PIR. Conversely, the 90% positive detection rate for samples stored at 25 °C was only achieved in the first week; the detection rate decreased with longer storage (Figure 4). The mean positive detected rates of the samples stored at three different temperatures were not significantly different between samples tested on the date of collection and those stored for 1 week (*p* = 0.92). After the samples were stored for 3, 5, 7, 9, 11, for 13 weeks, the mean positive detection rate of the 25 °C samples was lower than the rates of those stored at −20 °C and 4 °C (*p* < 0.05). Moreover, the detection rate of the 25 °C samples significantly decreased with longer storage (*p* < 0.0001), but no differences were found between those stored at −20 °C (*p* = 0.21) and 4 °C (*p* = 0.40) (Figure 5).

### 3.4. Trial 4: Odor-Adsorbed Filter Paper Trained Dogs Detecting Live RIFA

**Figure 6:**
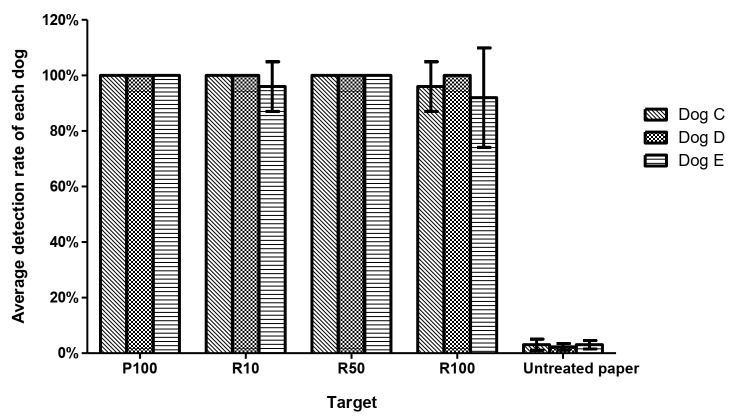
The RIFA detection rates of three dogs trained with odor-adsorbed filter paper. Columns represent the mean and standard deviation of 5 replicates of the tests performed by each detection dog. There were no differences in the positive detection rates in discriminating paper scented with 100 live RIFAs (P100) and 10, 50, and 100 live RIFAs (R10, R50, and R100) (*p* = 0.28, Kruskal–Wallis test). The average false response rate was 3.0%.

Dogs C, D, and E were trained in the present study and all achieved 100% PIR. They were then tested with 10, 50, and 100 live RIFAs. Their respective mean PIRs were 100%, 99%, 100%, and 96% in detecting paper scented with 100 live RIFAs and 10, 50, and 100 live RIFAs, with a mean false response rate of 3.0% (Figure 6). There were no differences among the positive detection rates in discriminating paper scented with 100 live RIFAs and 10, 50, and 100 live RIFAs (*p* = 0.28).

### 3.5. Trial 5: Field Trial—RIFA Detection in Culverts

Scented samples from RIFA-invaded underground wire culverts at Taoyuan International Airport were collected and sent for double-blind odor recognition. The mean PIR of dogs A, B, C, and E asked to identify RIFA-scented paper collected from the target culvert row was 87.5%, and the average false response was 7.8%. There were no significant differences among the dogs’ capabilities in discriminating the target (*p* = 0.68) and non-target samples (*p* = 0.76).

## 4. Discussion

Given their great capability for scent recognition, detection dogs play significant roles in locating both harmful insects and threatened and rare species. They have been utilized to detect invasive plants and animals such as the spotted knapweed (*Centaurea stoebe*), small Indian mongoose (*Herpestes javanicus*), and brown tree snake (*Boiga irregularis*) [26,36,37,38].

In the present study, two live RIFA-experienced dogs did not show different capabilities for detecting 10 live RIFAs in tubes, tubes with a filter paper scented by 100 live RIFAs for 4 h, or tubes with untreated paper. In addition, there were no significant differences in scent-detection capability (both PIR and false response rate) among the five dogs based on the data in trials 2, 3, 4, or 5. These results indicate a high ability of dogs trained for RIFA scent detection; all animals met the minimum requirement with a PIR of at least 90% and a false negative positive rate ≤10% [27].

Several tools have been used as odor adsorbents and transmitters, and as training aids for canine detection training [39]. Recently, more canine training aids were studied, such as odor capture-and-release materials for explosive odorants and ovarian cancer cell lines for ovarian cancer detection [40,41]. The gypsy moth (*Porthetria dispar* L.) sex attractant pheromone disparlure has been applied to different objects for training, and dogs’ capacity to retrieve disparlure-treated sticks was higher than for treated rubber balls or tin cans [31]. Exudates from screwworm-infested wounds placed on balls and larvae-scented balls were used as target samples for dog training [32]. For termite detection, dogs were taught to associate terry cloth towels with termite scent [27]. A filter paper impregnated with bed bug scent using pentane extraction was 100% indicated by trained dogs and therefore useful for detector training [29]. Despite these reports, there was no scientific evidence validating the utility of filter paper odor adsorption. The present study is the first to show that it is a competent odor transporting medium.

Carpet squares have been used as a scent carrier of residual human odor in forensic studies. Three well-trained cadaver dogs displayed excellent validity, predictive value, and accuracy for identifying positive samples [42]. More recently, grave soil was reported to hold residual human odor and can be recognized by properly trained dogs [43]. Commercially available pseudoscents from Sigma-Aldrich (St. Louis, MO, USA) have also been applied as canine training aids to facilitate the detection of corpses and narcotics such as cocaine, heroin, and marijuana. In the field of human forensic science, cotton napkin (ARATEX™, Chlumtex) have been used in routine casework [44]. However, studies on scent carriers for RIFAs are still rare, and the stability of scent samples used in animal chemosignaling was possibly violated during analytical procedures by storage protocols, storage containers, light exposure, storage time and different temperature treatments [45,46].

RIFAs produce several semiochemicals such as venom alkaloids, cuticular hydrocarbons, and trail pheromones [47]. In the present study, dogs were trained to recognize the odor of RIFAs on filter paper. The carried scent may contain various organic compounds, and further research is necessary to determine exactly what the trained dogs smelled.

Detection dogs have been utilized to detect the invasive brown tree snake (*Boiga irregularis*). This reptile has substantially reduced or even eradicated native animal populations, but on Guam it threatened electrical utilities and human safety [36]. Given the risk of snakes on Guam, the government of Hawaii imported one live, sterile male brown tree snake to train its detection dogs. To secure this invasive damaging species, a radio transmitter was implanted into the snake, and a double-secured container and snake-catching equipment were used for restraint. Live fire ants should not be imported into an RIFA-free zone, and are a biosecurity risk. For example, RIFA colonization in Japan has not been confirmed; however, RIFAs have been reported in 37 locations in 14 prefectures in Japan and mainly entered by the shipping containers imported from southern China [48]. Our study suggests a practical method to train detection dogs without introducing live RIFAs and a quick and safe way to correctly identify RIFA-invaded zones. The results demonstrate that filter paper is a useful aid for detection dog training in uninvaded areas. Moreover, filter paper can be used to collect and transmit other odors for dog training. Future studies on the capacity of scented filter papers transported by plane will be necessary since air pressure changes with altitude.

According to the legislation of the Taiwanese Ministry of Transportation and Communication, Civil Aeronautics Administration, dogs are not allowed to enter restricted areas in airports for RIFA searching. Our results suggest that detection dogs trained with filter paper have a good positive detection rate and low false response rate. Therefore, this method can be applied to train dogs to detect RIFAs in quarantined or segregated areas. Detection dogs might not be able to enter mechanical facilities and go near electrical equipment; in these scenarios, filter paper could be used to adsorb the scent and then sent for dog interpretation. This approach would minimize risk to detecting dogs and save manpower, time, and money.

Using canine detectors for RIFAs is more effective than other methods of detection when the ants are at low density [35]. The results of using canine detectors and RIFA-scented filter paper in the present investigation reveal a practical method on RIFA detection for regions or countries where fire ants have not invaded. The use of RIFA-scented papers as an odor reservoir for dog training could increase detection efficiency, achieve economic savings, and decrease the risk of RIFA introduction into uninvaded or eradicated areas.

## Figures and Tables

**Table 1:** Canine demographics.

Name	Breed	Sex	Age	Inspection Experience
Dog A	Beagle	Male (neutered)	7	Red imported fire ant
Dog B	Beagle	Male (neutered)	5	Red imported fire ant
Dog C	Beagle	Female	3	None
Dog D	Beagle	Female (spayed)	3	None
Dog E	Beagle mix	Female (spayed)	4	None

**Table 2:** The positive indication rates for filter papers scented by 10, 50, and 100 RIFAs for 4 h.

Dog	Positive Indication Rate			False Positive Rate
	P100 *	P50 *	P10 *	
A	92.0 ± 11.0%	100.0 ± 0.0%	92.0 ± 11.0%	0.8 ± 0.9%
B	96.0 ± 8.9%	92.0 ± 11.0%	100.0 ± 0.0%	2.9 ± 3.9%
C	100.0 ± 0.0%	96.0 ± 8.9%	100.0 ± 0.0%	2.7 ± 2.9%
D	92.0 ± 11.0%	84.0 ± 26.0%	92.0 ± 11.0%	2.0 ± 1.0%
E	88.0 ± 11.0%	100.0 ± 0.0%	92.0 ± 11.0%	0.8 ± 0.5%
Mean	93.6 ± 9.5%	94.4 ± 13.6%	95.2 ± 8.7%	2.0 ± 2.0%

* Positive indication rate ± SD.

## Data Availability

The data presented in this study are available on request from the corresponding author.
